# Risk Factors for Mortality among Patients with *Pseudomonas aeruginosa* Bloodstream Infections: What Is the Influence of XDR Phenotype on Outcomes?

**DOI:** 10.3390/jcm9020514

**Published:** 2020-02-14

**Authors:** María Milagro Montero, Inmaculada López Montesinos, Hernando Knobel, Ema Molas, Luisa Sorlí, Ana Siverio-Parés, Nuria Prim, Concepción Segura, Xavier Duran-Jordà, Santiago Grau, Juan Pablo Horcajada

**Affiliations:** 1 Infectious Diseases Service, Hospital del Mar, Infectious Pathology and Antimicrobials Research Group (IPAR), Institut Hospital del Mar d’Investigacions Mèdiques (IMIM), Universitat Autònoma de Barcelona (UAB), CEXS-Universitat Pompeu Fabra, Spanish Network for Research in Infectious Diseases (REIPI), 08003 Barcelona, Spain; 95422@parcdesalutmar.cat (M.M.M.); 87138@parcdesalutmar.cat (H.K.); emamolas@hotmail.com (E.M.); lsorli@hospitaldelmar.cat (L.S.); jhorcajada@psmar.cat (J.P.H.); 2 Microbiology Service, Laboratori de Referència de Catalunya, Hospital del Mar, 08820 Barcelona, Spain; siverio.a@gmail.com (A.S.-P.); nuriaprim@gmail.com (N.P.); conchasegur@hotmail.com (C.S.); 3 Methodology and Biostatistics Support Unit, Institut Hospital del Mar d’Investigacions Mèdiques (IMIM), 08003 Barcelona, Spain; xduran@imim.es; 4 Pharmacy Service, Hospital del Mar, Infectious Pathology and Antimicrobials Research Group (IPAR), Institut Hospital del Mar d’Investigacions Mèdiques (IMIM), Universitat Autònoma de Barcelona (UAB), CEXS-Universitat Pompeu Fabra, 08003 Barcelona, Spain; sgrau@parcdesalutmar.cat

**Keywords:** extensively drug-resistant Pseudomonas aeruginosa, multidrug resistance, high-risk clones, combined antimicrobial therapy, bacteremia, outcome

## Abstract

This study aimed to assess the impact of extensively drug-resistant (XDR) phenotype on mortality in *Pseudomonas aeruginosa* bacteremia. A retrospective cohort study was performed in a tertiary hospital from January 2000 to December 2018. All consecutive prospectively recorded *P. aeruginosa* bacteremia in adult patients were assessed. In this study, 382 patients were included, of which 122 (31.9%) due to XDR *P. aeruginosa*. Independent factors associated with 14-day mortality were as follows: high-risk source of bacteremia (hazard ratio (HR) 3.07, 95% confidence interval (CI), 1.73–5.46), septic shock (HR 1.75, 95% CI, 1.12–2.75), and higher Pitt scores (one-point increments; HR 1.25, 95% CI, 1.12–1.38). Otherwise, the appropriateness of definitive antibiotic therapy was a protective factor (HR 0.39, 95% CI, 0.24–0.62). The same variables were also associated with 30-day mortality. XDR phenotype was not associated with 14- or 30-day mortality. In a subanalysis considering only high-risk source cases, combined antimicrobial therapy was independently associated with 14-day favorable outcome (HR 0.56, 95% CI, 0.33–0.93). In conclusion, XDR phenotype was not associated with poor prognosis in patients with *P. aeruginosa* bacteremia in our cohort. However, source of infection, clinical severity, and inappropriate definitive antibiotic therapy were risk factors for mortality. Combined antimicrobial therapy should be considered for high-risk sources.

## 1. Introduction

*Pseudomonas aeruginosa* is one of the most difficult-to-treat microorganisms due to its intrinsic resistance profile and its extraordinary ability to develop additional resistance through selection of chromosomal mutations and acquisition of resistance genes [1,2]. In recent years, the emergence of high-risk clones that select multidrug- (MDR) or extensively drug-resistant (XDR) strains widely disseminated in hospitals throughout the world is also a factor of concern [2,3,4]. The increase in MDR/XDR strains seriously compromises antibiotic treatment options and consequently the probability of receiving appropriate early antimicrobial drugs, which may lead to poor outcomes, particularly in the presence of severe infections [5,6,7].

*P. aeruginosa* has an extraordinary capacity for causing a wide range of infections. Bloodstream infection (BSI) is considered one of its most serious and dreaded complications, with a reported mortality ranging from 18% to 61% [8]. It has been hypothesized that BSIs caused by antimicrobial-resistant strains lead to worse outcomes than those caused by susceptible ones, although controversial findings have been reported over the years [9,10,11,12,13,14,15,16]. These conflicting results could partly be due to the difficulty of elucidating the influence of other factors on outcomes, such as underlying conditions, infectious syndrome severity, source of infection, therapeutic management, or bacterial virulence determinants [9,10,11,12,13,14,15,16,17]. Those studies have some limitations: some results were obtained before Magiorakos et al. [5] standardized the terminology, or included different sources of infection, or were limited by insufficient sample sizes, and reliable conclusions could not be drawn. Furthermore, most were focused only on carbapenem-resistant or MDR *P. aeruginosa* strains, but not specifically on XDR isolates, which have only few active drugs available and are more prone to be linked to high-risk clones. In line with this, previous studies showed that almost all XDR isolates analyzed at our institution belonged to well-described *P. aeruginosa* high-risk clones, with sequence type 175 (ST175) being by far the most frequent high-risk clone observed [18,19,20,21]. Unpublished local data also showed that 85% of our XDR *P. aeruginosa* isolates were clonally related, showing an endemic situation at our center.

Hence, we present a retrospective analysis of a large cohort of *P. aeruginosa* bacteremia designed to assess the impact of XDR phenotype on mortality.

## 2. Methods

### 2.1. Hospital Setting, Study Design, and Participants

The study was conducted at the Hospital del Mar, a 420-bed tertiary-care university hospital in Barcelona, Spain. We performed a retrospective analysis of all patients aged ≥ 18 years old that had been prospectively recorded with positive blood cultures for *P. aeruginosa* from January 2000 to December 2018. Positive blood cultures were reported daily by the Microbiology Department. Patients were followed for up to 30 days from the onset of BSI to assess 14- and 30-day all-cause mortality. Only the first episode of bacteremia per each patient was considered. Polymicrobial BSIs were excluded.

The study was approved by the Clinical Research Ethics Committee of Parc de Salut (register no. 2019/8758I). The need for written informed consent was waived due to the observational nature of the study and retrospective analysis.

### 2.2. Study Objectives and Outcomes

We aimed to assess the impact of XDR phenotype on patients with *P. aeruginosa* bacteremia. The main outcomes were mortality at 14 and 30 days after the onset of bacteremia. An additional subanalysis was performed involving only the patients with high-risk sources of infection.

### 2.3. Variables and Data Source

Trained study investigators collected demographic, clinical, and microbiological data from the hospital or primary care electronic medical charts and the microbiology laboratory database, including the following: sex, age, underlying diseases, site of acquisition, source of infection, severity of BSI, antibiotic treatment, BSI microbiological resistance profile, and 14- and 30-day all-cause mortality. Follow-up information for up to 30 days after the onset of bacteremia was obtained by reviewing electronic medical charts.

### 2.4. Microbiological Studies

Bacterial growth in blood cultures was detected by the BacT/Alert^®^ System (bioMérieux, Marcy l’Etoile, France). Bacterial identification was performed by standard biochemical tests and by MALDI-TOF since 2014. Antibiotic susceptibility testing was routinely performed by two dilution-based methods in two different periods due to changes in the laboratory workflow: broth microdilution panels MicroScan^®^ using the WalkAway^®^ system (Beckman Coulter, Brea, California, United States) from 2000 to 2008, and AST cards using the VITEK^®^2 System (bioMérieux, Marcy l’Etoile, France) from then on. Antimicrobials tested were the following: ciprofloxacin, piperacillin-tazobactam, ceftazidime, cefepime, imipenem, meropenem, aztreonam, gentamicin, tobramycin, amikacin. and colistin. Antimicrobial susceptibility testing results were categorized according to the Clinical and Laboratory Standards Institute (CLSI) until 2013, and thenceforth according to the European Committee on Antimicrobial Susceptibility Testing (EUCAST). Isolates categorized as intermediate were considered as resistant.

*P. aeruginosa* isolates were classified as XDR when they were non-susceptible to at least one agent in all but two or fewer antipseudomonal antimicrobial categories, and as MDR when they were non-susceptible to at least one agent in three or more antipseudomonal antimicrobial categories [5]. Those strains that did not meet the previous criteria were classified as non-MDR *P. aeruginosa*. Both non-MDR and MDR isolates were included in the non-XDR group.

Clonality and antibiotic resistance mechanisms were not investigated specifically for this study, but some strains had been analyzed by our group before [18,20,21]. As previously reported in detail, clonal relatedness of XDR *P. aeruginosa* isolates was analyzed using pulsed-field gel electrophoresis, multilocus sequence typing, and whole-genome sequencing. Regarding resistant mechanisms, overexpression of chromosomal β-lactamase AmpC or efflux pumps, OprD deficiency, or horizontal acquired enzymes were investigated, among others [18,20,21].

### 2.5. Definitions

*P. aeruginosa* bacteremia was defined as at least one positive blood culture obtained from a patient with signs and symptoms of infection.

Presence of comorbidities and severity of underlying diseases were assessed by the Charlson comorbidity index [22] and McCabe score [23]. Neutropenia was defined as an absolute neutrophil count of ≤500 cells/mm^3^. Site of acquisition was defined according to the classification by Friedman et al. [24]. BSIs that did not meet the nosocomial or healthcare-associated criteria were considered as of community acquisition. BSI severity was evaluated by Pitt score [25], the need of intensive care unit admission, and the presence of septic shock, defined as the need for vasopressors to maintain a mean arterial pressure of at least 65 mmHg [26]. Source of infection was defined as the most likely origin of infection responsible for the BSI according to medical records. Catheter-related bloodstream infection, urinary tract infection, respiratory tract infection, skin and soft tissue infection, intraabdominal infection, and other sources (including endocarditis, otorhinolaryngologic, central nervous system, and surgical site sources) were included. When the origin of the infection was unclear, it was defined as BSI of unknown origin. For risk of mortality [10], sources of infection were divided into two groups: (a) low-risk sources or those involving either catheter-related bloodstream infections or urinary tract infections and (b) high-risk sources or those involving others.

Appropriate empiric antibiotic therapy was considered when at least 1 antipseudomonal antibiotic with in vitro activity was administered during the first 24 h after taking the blood cultures, whereas appropriate definite antibiotic therapy refers to the moment of knowing bacteria susceptibility results. When pneumonia was the source of infection, aminoglycoside monotherapy was considered an inadequate treatment [27]. Combination therapy was defined as two antipseudomonal drugs used for at least 24 h.

### 2.6. Statistical Analysis

Continuous quantitative variables were presented as median and interquartile range (IQR). For categorical variables, number of cases and percentages were used. The Student’s *t*-test or Mann–Whitney U test were applied to compare continuous variables, and Fisher’s exact test or Pearson’s χ^2^ test to contrast categorical variables, as appropriate. The Cox proportional hazards model was used to explore 14- and 30-day mortality events. Univariate and multivariate survival analyses were performed, and results were reported as the hazard ratio (HR) and 95% confidence interval (CI). The proportional hazard assumption checked by examining Schoenfeld residuals (for overall model and variable-by-variable) was not violated. The variables introduced into the multivariate analysis included those with a crude *p* value ≤ 0.1 in the univariate analysis or those considered clinically relevant for the outcome and the study hypothesis. The explained criteria are based on backward selection but controlling by clinical significance. All *p*-values were two-tailed and statistical significance was <0.05. Statistical analyses were performed using STATA 15.1.

## 3. Results

**Figure 1:**
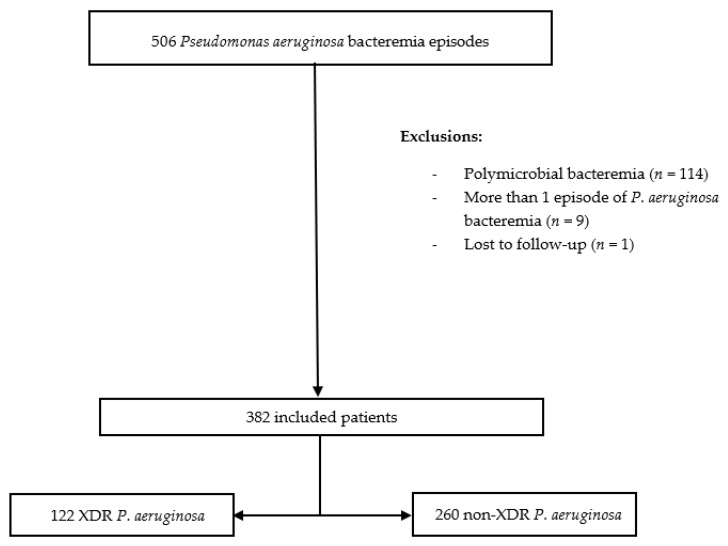
Flowchart of the patients included in the study.

A total of 506 episodes of *P. aeruginosa* bacteremia were assessed during the study period. Of these, 124 were excluded: 114 had polymicrobial bacteremia, 9 patients presented more than one episode of *P. aeruginosa* BSI, and another was lost to follow-up. Therefore, 382 patients were finally recorded, and 122 (31.9%) of these episodes corresponded to XDR *P. aeruginosa* (Figure 1). In the non-XDR group, 35/260 (13.5%) were MDR and 225/260 (86.5%) non-MDR strains.

**Figure 2:**
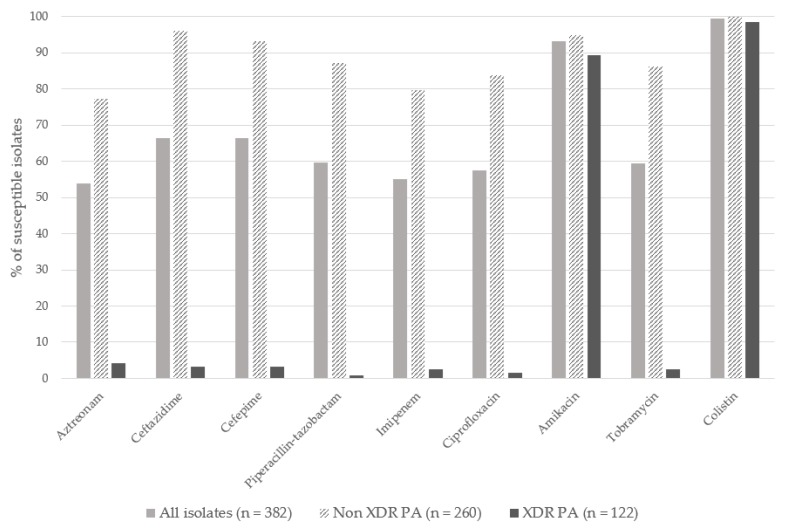
Susceptibility to different antimicrobial agents of the 382 isolates of *Pseudomonas aeruginosa.*

The rate of antimicrobial susceptibility of the total cohort and according to XDR pattern are shown in Figure 2. With respect to the XDR antimicrobial susceptibility profile, the most common pattern observed was non-susceptibility to all antipseudomonal agents except colistin (98.4%, *n* = 120/122) and amikacin (89.3%, *n* = 109/122).

Demographics and clinical characteristics of the total cohort and according to XDR pattern are shown in Table 1. Compared to patients with non-XDR *P. aeruginosa* bacteremia, those with XDR *P. aeruginosa* were more likely to have a low-risk source of bacteremia (50.8% vs. 35.8%, *p* = 0.005), but a higher Pitt score (median points, 2 (IQR 1 to 4) vs. 2 (IQR 0 to 3); *p* = 0.017). Among patients with XDR bacteremia, the following significant differences were also observed when compared with non-XDR BSI episodes: history of hematologic malignancy (22.1% vs. 11.9%; *p* = 0.01), nosocomial acquisition (82.8% vs. 55%; *p* < 0.001), receiving inappropriate empirical antibiotic therapy (88.5% vs. 42.3%; *p* < 0.001), and use of combined antimicrobial therapy (51.6% vs. 33.5%; *p* < 0.001).

### 3.1. Mortality and Risk Factors for Mortality

The 14-day all-cause mortality rate for all patients was 23.3% (89/382), whereas at day 30, it was 30.9% (118/382). No statistically significant differences in mortality rates were found between XDR and non-XDR *P. aeruginosa* groups at either day 14 or day 30. 

Multivariate analysis using a Cox proportional hazards model, adjusted for sex and age, showed that the independent risk factors for 14-day all-cause mortality were as follows: a high-risk source of bacteremia (HR 3.07, 95% CI, 1.73 to 5.46; *p* < 0.001), presentation with septic shock (HR 1.75, 95% CI, 1.12 to 2.75; *p* = 0.015), and higher Pitt scores (one-point increments; HR 1.25, 95% CI, 1.12 to 1.38; *p* < 0.001). Receiving appropriate definitive antimicrobial therapy was a protective factor (HR 0.39, 95% CI, 0.24 to 0.62; *p* < 0.001). XDR *P. aeruginosa* BSI was not related to 14-day mortality (HR, 1.07; 95% CI, 0.68 to 1.67; *p* = 0.777) (Table 2).

Regarding 30-day mortality, the independent risk factors for mortality observed in a Cox proportional hazards model, adjusted for sex and age, were as follows: a high-risk source of infection (HR 2.49, 95% CI, 1.56 to 3.99; *p* < 0.001), septic shock (HR 1.77, 95% CI, 1.18 to 2.65; *p* = 0.006), and Pitt scores (one-point increments; HR 1.25, 95% CI, 1.13 to 1.37; *p* < 0.001), whereas appropriate definitive therapy (HR 0.42, 95% CI, 0.27 to 0.66; *p* < 0.001) was identified as a protective factor. XDR *P. aeruginosa* BSI was not related to 30-day mortality (HR 1.14, 95% CI, 0.77 to 1.69; *p* = 0.504) (Table 3).

### 3.2. Subanalysis

Because the XDR group more frequently had a low-risk source of bacteremia, an additional analysis was performed that considered only high-risk sources of BSI (Table S1, Supplementary Materials). In multivariate analysis, factors independently associated with 14- and 30-day all-cause mortality were once again Pitt score, septic shock, and inappropriate definitive antibiotic therapy. In addition, at day 14, combined antimicrobial therapy was identified as a protective factor for mortality (HR 0.56, 95% CI 0.33 to 0.93; *p* = 0.027) (Tables S2 and S3, Supplementary Materials).

## 4. Discussion

Multiple reports have drawn attention to the worryingly high rates of XDR *P. aeruginosa* infections worldwide [1,2,28,29]. Although previous studies have assessed the impact on the outcome of carbapenem-resistant or MDR *P. aeruginosa* BSI [9,10,11,12,13,14,15,16], little is known about the role of XDR strains. In order to obtain a better understanding of this issue, we present here the results of the largest cohort of patients with monomicrobial XDR *P. aeruginosa* bacteremia published to date.

Our data show that the vast majority of XDR *P. aeruginosa* strains were non-susceptible to all antipseudomonal agents apart from colistin and amikacin. Although clonality and the resistance mechanisms of the isolates were not specifically investigated in this study, previous ones found that, in XDR *P. aeruginosa* strains, the ST175 high-risk clone was by far the most frequent at our site [18,19,20,21]. In a Spanish multicenter study by our group (COLIMERO study) [18], 17/21 (81%) strains included from our center belonged to the ST175 high-risk clone. In another recent survey of *P. aeruginosa* molecular epidemiology and antimicrobial resistance in Spain, del Barrio-Tofiño et al. [19] found that all isolates recovered from our site also belonged to the ST175 high-risk clone. In these two previous reports [18,19], the underlying resistance mechanisms in the XDR phenotype were mainly a combination of chromosomal mutations such as hyperproduction of chromosomal AmpC β-lactamases, efflux pumps, OprD deficiency and/or quinolone resistance-determining region mutations, rather than horizontally acquired carbapenemases.

Our study showed that the all-cause mortality rate for patients with XDR *P. aeruginosa* bacteremia at day 30 was above 30%, which is similar to results reported in previous series of carbapenem-resistant or MDR isolates [9,10,13,16,17,30]. We observed that severity at presentation or a high-risk source of *P. aeruginosa* bacteremia were independent predictors of mortality, as has been previously reported [9,10,12,14,15,16]. On the other hand, although it is often assumed that antibiotic-resistant *P. aeruginosa* bacteremia results in worse outcomes, our study found that an XDR profile was not associated with higher mortality rates, even after considering only high-risk source BSI. Conflicting results have been reported for carbapenem-resistant or MDR *P. aeruginosa* infections [9,10,11,12,13,14,15,16]. In the case of XDR isolates, the evidence is even scarcer and there are only a few published studies. Recio et al. [31] also failed to identify the XDR phenotype as a predictor of mortality in a cohort of 70 patients with *P. aeruginosa* bacteremia in Spain that included 24 XDR strains (10 of them belonging to the ST175 high-risk clone and 11 to ST235). Another study carried out in Brazil [11] did not find an association in 120 patients with *P. aeruginosa* bacteremia, 31 of them XDR strains. On the other hand, a recent study of a cohort of 243 patients, including 87 XDR isolates (43 strains linked to ST175 high-risk clone and 33 to ST235) [16] found MDR phenotype as an independent predictor of 30-day crude mortality. In line with this, a recent Spanish study aimed at assessing the impact of virulence on the outcome of *P. aeruginosa* BSI [32] also found that XDR phenotype was statistically linked to 30-day mortality in a cohort of 593 bacteremia cases, 81 of them XDR isolates (61 ST175, 9 ST111, 2 ST235, and 2 ST244), although multivariate analysis data were not provided. Another Spanish study [33] focused on BSIs in solid organ transplant recipients identified a higher mortality rate for patients with bacteremia due to XDR *P. aeruginosa* (*n* = 31) compared to those with BSI caused by other microorganisms (*n* = 287), and it was found to be an independent risk factor for mortality (20/22 studied strains showed the ST175 pattern). Other studies [34,35] have addressed the impact of XDR strains on different sources of infection, not exclusively in bacteremia. For instance, a Thai study of 255 patients with *P. aeruginosa* infections, including 56 XDR strains, found that an XDR phenotype was an independent factor of mortality attributable to infection when compared to non-XDR isolates [34]. Samonis et al. found the same association with mortality in a cohort of 89 *P. aeruginosa* infections (52 of them with bacteremia and 22 XDR isolates) in cancer patients in Greece [35].

As was the case for carbapenem-resistant or MDR *P. aeruginosa* bacteremia, conflicting findings are also reported for XDR strains, suggesting that the prognosis of BSI depends on several factors apart from the resistance phenotype. In our study, no relevant differences were observed between the two groups either for preexisting comorbidities or severity of clinical presentation at the onset of bacteremia, although a low-risk source of bacteremia was more frequently observed in the XDR group. This finding could partially explain the lack of association between XDR phenotype and mortality, although the same results were obtained after adjusting for source of infection, which rules out this hypothesis. In addition, although patients with an XDR *P. aeruginosa* BSI have a higher probability of receiving inadequate empirical antimicrobial therapy, unexpectedly, this was not associated with worse outcomes. Some authors argue that in cases of *P. aeruginosa* bacteremia, a 48–72 h delay before receiving appropriate antibiotics may not be so crucial to patient outcome, since mortality is mainly due to other factors, such as severity of infection, having a high-risk source, or receiving inappropriate definitive antibiotic therapy [10,12,15,36,37], which is consistent with the results of the present study.

Another hypothesis to explain the lack of impact of XDR phenotype on mortality could be the influence of the acquisition of resistance mechanisms. It has been hypothesized that it may involve a fitness cost resulting in strains with lower virulence, and with a reduced inflammatory response in the host when compared to susceptible isolates [38,39,40,41]. On the other hand, it has also been reported that not all resistance mutations lead to a biological cost such as the OprD deficiency [42]. Indeed, some studies have identified that MDR strains can develop compensatory or suppressor mechanisms that allow them to recover their baseline fitness [12,43,44]. Other studies have found that some high-risk clones can be as virulent as susceptible strains, despite exhibiting many resistance mutations, suggesting that pathogenicity depends not only on the fitness cost of resistance, but also on the presence of certain virulence determinants such as exoU-positive genotype or O11 antigen serotype [16,17,31,32]. The ST235 high-risk clone, for example, appears to be particularly virulent in cases of ExoU production, whereas the virulence of ST175, the most prevalent high-risk clone observed at our institution, is especially low [16,17,31,32]. Thus, it may be inferred that the high prevalence of the less virulent ST175 high-risk clone at our institution may account in part for the lack of detrimental impact on mortality of the XDR phenotype.

One of the most interesting findings of our study is that combined antimicrobial therapy improves prognosis at day 14 in high-risk source bacteremia. Previous studies have addressed this issue [45,46,47,48,49], although they were not focused specifically on XDR *P. aeruginosa* bacteremia. Some of these [46,47,48] also found a protective effect for patients receiving two active drugs for high-risk sources, mainly pneumonia. In our study, lower respiratory tract infection was the most common high-risk source of infection, closely followed by primary bacteremia. Nevertheless, this finding should be interpreted with caution. As this issue was not one of the main purposes of our research, certain crucial variables, such as the duration of combined therapy, the onset time of this therapy, the antibiotic dosing, or how antibiotic treatment was administered (extended- or continuous-infusion or intermittent bolus) were not accounted for. Further studies are needed to clarify this question.

This study has several limitations. First, although cases were recorded prospectively, several data were obtained retrospectively and are therefore more vulnerable to potential bias. Second, the study was carried out in a single center and the results are not necessarily transferable to other settings with a different epidemiology. Third, although a sample of the included isolates was well characterized in previous studies, clonality and resistance mechanisms were not specifically investigated in all cases. Finally, some relevant variables such as the time until appropriate definitive antibiotic treatment or those related to combined antimicrobial therapy were not recorded, thus their impact on outcomes could have been potentially overlooked. On the other hand, this study has some highlighted strengths. It includes the largest published sample size of XDR *P. aeruginosa* bacteremia, eliminating the biases of many studies with small sample sizes, which reduce the statistical power to be able to draw reliable conclusions. Furthermore, a wide-ranging statistical analysis was performed to control for confounding variables, including a subanalysis in high-risk sources.

## 5. Conclusions

Our study identified severity at presentation, having a high-risk source of bacteremia, and inappropriate definitive antibiotic therapy as risk factors for mortality in patients with *P. aeruginosa* bacteremia. On the other hand, the XDR phenotype was not associated with poor prognosis. Decreased virulence in XDR strains, theoretical fitness costs, and a high prevalence of the less virulent ST175 high-risk clone at our institution may be among the reasons for these findings. Nevertheless, the mortality rate for *P. aeruginosa* bacteremia remains high in our cohort. Since antibiotic treatment is the only modifiable factor to try to improve outcome, strategies aimed at earlier identification of patients with risk factors for *P. aeruginosa* bacteremia should be implemented to avoid delays in the administration of effective antibiotic therapy, mainly in more severe patients and those with high-risk source bacteremia. Combined antimicrobial therapy should be considered in patients with bacteremia from high-risk sources.

## Figures and Tables

**Table 1:** Baseline characteristics of the patients. Data are presented as *n* (%), unless otherwise specified. Abbreviations: m (median), IQR (interquartile range), XDR PA (extensively drug-resistant *Pseudomonas aeruginosa*), ICU (intensive care unit).

Variable	All Episodes (*n* = 382)	Non-XDR PA (*n* = 260)	XDR PA (*n* = 122)	*p*-Value
Demographic information				
Age in years, m (IQR)	70.5 (60–78)	72 (60.25–78)	69 (59–76)	0.219
Male sex	276 (72.3)	190 (73.1)	86 (70.5)	0.599
Nosocomial acquisition	244 (63.9)	143 (55)	101 (82.8)	<0.001
Underlying condition				
Diabetes mellitus	94 (24.6)	63 (24.2)	31 (25.4)	0.803
Chronic obstructive pulmonary disease	82 (21.6)	53 (20.5)	29 (23.8)	0.475
Cirrhosis	28 (7.3)	18 (6.9)	10 (8.2)	0.656
Hemodialysis	25 (6.5)	18 (6.9)	7 (5.7)	0.662
Hematologic malignancy	58 (15.2)	31 (11.9)	27 (22.1)	0.010
Solid tumor malignancy	144 (37.7)	102 (39.2)	42 (34.4)	0.366
Neutropenia	70 (18.3)	51 (19.6)	19 (15.6)	0.341
Charlson comorbidity index, m (IQR)	4 (2–6)	4 (2–6)	4 (3–6)	0.911
McCabe score				
Non-fatal McCabe	107 (28)	73 (28.1)	34 (27.9)	0.966
Rapidly fatal McCabe	117 (30.6)	86 (33.1)	31 (25.4)	0.130
Ultimately fatal McCabe	158 (41.4)	101 (38.8)	57 (46.7)	0.145
Source of infection				
Catheter-related bloodstream infection	39 (10.2)	19 (7.3)	20 (16.4)	0.006
Urinary tract infection	116 (30.4)	74 (28.5)	42 (34.4)	0.237
Respiratory infection	95 (24.9)	67 (25.8)	28 (23)	0.552
Skin and soft tissue infection	19 (5)	15 (5.8)	4 (3.3)	0.297
Intraabdominal infection	45 (11.8)	36 (13.8)	9 (7.4)	0.067
Primary or Unknown	62 (16.2)	45 (17.3)	17 (13.9)	0.404
Other	6 (1.6)	4 (1.5)	2 (1.6)	1
High-risk source	227 (59.4)	167 (64.2)	60 (49.2)	0.005
Low-risk source	155 (40.6)	93 (35.8)	62 (50.8)	0.005
Baseline illness severity				
Pitt score, m (IQR)	2 (1–3)	2 (0–3)	2 (1–4)	0.017
Pitt score ≥ 2	206 (53.9)	133 (51.2)	73 (59.8)	0.112
Septic shock	103 (27)	65 (25)	38 (31.1)	0.207
ICU admission	96 (25.1)	61 (23.5)	35 (28.7)	0.272
Antibiotic management				
Appropriate empirical treatment	164 (42.9)	150 (57.7)	14 (11.5)	<0.001
Appropriate definitive treatment	332 (86.9)	227 (87.3)	105 (86.1)	0.737
Combined antimicrobial therapy	150 (39.3)	87 (33.5)	63 (51.6)	0.001
All-cause mortality				
Day 14	89 (23.3)	59 (22.7)	30 (24.6)	0.682
Day 30	118 (30.9)	77 (29.6)	41 (33.6)	0.431

**Table 2:** Univariate and multivariate Cox model of 14-day all-cause mortality. Data are presented as *n* (%), unless otherwise specified. Abbreviation: HR (hazard ratio), CI (confidence interval), m (median), IQR (interquartile range), ICU (intensive care unit), XDR PA (extensively drug-resistant *Pseudomonas aeruginosa*), BSI (bloodstream infection).

Variables	Alive (*n* = 293)	Death (*n* = 89)	Crude HR (95% CI)	*p*-Value	HR Multivariate (95% CI)	*p*-Value
Demographic information						
Age in years, m (IQR)	70 (59.5–78)	71 (62.5–78)	1.01 (0.99–1.02)	0.252	1.01 (0.99–1.03)	0.119
Male sex	209 (71.3)	67 (75.3)	1.17 (0.72–1.89)	0.518	1.12 (0.69–1.82)	0.645
Nosocomial acquisition	186 (63.5)	58 (65.2)	1.05 (0.69–1.4)	0.796		
Underlying condition						
Diabetes mellitus	73 (24.9)	21 (23.6)	0.96 (0.59–1.56)	0.858		
Chronic obstructive pulmonary disease	57 (19.6)	25 (28.1)	1.49 (0.94–2.37)	0.09		
Cirrhosis	21 (7.2)	7 (7.9)	1.09 (0.51–2.37)	0.814		
Hemodialysis	22 (7.5)	3 (3.4)	0.47 (0.15–1.48)	0.196		
Hematology malignancy	42 (14.3)	16 (18)	1.22 (0.71–2.1)	0.465		
Solid tumor malignancy	114 (38.9)	30 (33.7)	0.81 (0.52–1.26)	0.354		
Neutropenia	51 (17.4)	19 (21.3)	1.22 (0.73–2.02)	0.445		
Charlson comorbidity index, m (IQR)	4 (2–6)	4 (2–6)	0.99 (0.93–1.07)	0.996		
McCabe score			1.16 (0.89–1.51)	0.249		
Non-fatal McCabe	83 (28.3)	24 (27)	0.98 (0.61–1.56)	0.932		
Rapidly fatal McCabe	96 (32.8)	21 (23.6)	0.67 (0.41–1.09)	0.104		
Ultimately fatal McCabe	114 (38.9)	44 (49.4)	1.41 (0.93–2.14)	0.105		
Origin of bacteremia						
Catheter-related bloodstream infection	37 (12.6)	2 (2.2)	0.18 (0.05–0.74)	0.017		
Urinary tract infection	103 (35.2)	13 (14.6)	0.36 (0.19–0.65)	0.001		
Respiratory infection	60 (20.5)	35 (39.3)	2.17 (1.42–3.32)	<0.001		
Skin and soft tissue infection	13 (4.4)	6 (6.7)	1.42 (0.62–3.25)	0.408		
Intraabdominal infection	35 (11.9)	10 (11.2)	0.94 (0.49–1.81)	0.852		
Primary or Unknown	40 (13.7)	22 (24.7)	1.84 (1.14–2.98)	0.013		
Other	5 (1.7)	1 (1.1)	0.69 (0.09–4.97)	0.715		
High-risk source	153 (52.2)	74 (83.1)	3.85 (2.17–6.67)	<0.001	3.07 (1.73–5.46)	<0.001
Low-risk source	140 (47.8)	15 (16.9)	0.26 (0.15–0.46))	<0.001		
Baseline illness severity						
Pitt score, m (IQR)	1 (0.5–2)	4 (1–4)	1.36 (1.24–1.49)	<0.001	1.25 (1.12–1.38)	<0.001
Pitt score ≥ 2	140 (47.8)	66 (74.2)	2.78 (1.73–4.48)	<0.001		
Septic shock	63 (21.5)	40 (44.9)	2.59 (1.71–3.95)	<0.001	1.75 (1.12–2.75)	0.015
ICU admission	56 (19.1)	40 (44.9)	2.92 (1.92–4.44)	<0.001		
Antibiotic management						
Appropriate empirical treatment	126 (43)	38 (42.7)	0.99 (0.65–1.51)	0.968		
Appropriate definitive treatment	266 (90.8)	66 (74.2)	0.35 (0.22–0.57)	<0.001	0.39 (0.24–0.62)	<0.001
Combined antimicrobial therapy	114 (38.9)	36 (40.4)	1.02 (0.67–1.88)	0.545		
XDR PA BSI	92 (31.4)	30 (33.7)	1.02 (0.68–1.56)	0.915	1.07 (0.68–1.67)	0.777

**Table 3:** Univariate and multivariate Cox model of 30-day all-cause mortality. Data are presented as *n* (%), unless otherwise specified. Abbreviation: HR (hazard ratio), CI (confidence interval), m (median), IQR (interquartile range), ICU (intensive care unit), XDR PA (extensively drug-resistant *Pseudomonas aeruginosa*), BSI (bloodstream infection).

Variables	Alive (*n* = 264)	Death (*n* = 118)	Crude HR (95% CI)	*p*-Value	HR Multivariate (95% CI)	*p*-Value
Demographic information						
Age in years, m (IQR)	70 (59–77.75)	72 (62.75–78)	1.01 (0.99–1.02)	0.159	1.01 (0.99–1.03)	0.052
Male sex	185 (70.1)	91 (77.1)	1.32 (0.85–2.04)	0.208	1.25 (0.8–1.95)	0.321
Nosocomial acquisition	162 (61.4)	82 (69.5)	1.31 (0.88–1.94)	0.178		
Underlying condition						
Diabetes mellitus	66 (25)	28 (23.7)	0.95 (0.62–1.46)	0.828		
Chronic obstructive pulmonary disease	50 (19)	32 (27.4)	1.47 (0.98–2.21)	0.062	1.1 (0.7–1.73)	0.675
Cirrhosis	19 (7.2)	9 (7.6)	1.06 (0.54–2.08)	0.876		
Hemodialysis	22 (8.3)	3 (2.5)	0.34 (0.11–1.06)	0.064	0.35 (1.01–1.15)	0.083
Hematology malignancy	38 (14.4)	20 (16.9)	1.15 (0.71–1.86)	0.575		
Solid tumor malignancy	102 (38.6)	42 (35.6)	0.88 (0.6–1.28)	0.505		
Neutropenia	48 (18.2)	22 (18.6)	1.03 (0.65–1.64)	0.884		
Charlson comorbidity index, m (IQR)	4 (2–6)	4 (2–6)	1.01 (0.95–1.07)	0.744		
McCabe score			1.2 (0.96–1.51)	0.116		
Non-fatal McCabe	77 (29.2)	30 (25.4)	0.89 (0.59–1.35)	0.596		
Rapidly fatal McCabe	87 (33)	30 (25.4)	0.72 (0.48–1.09)	0.124		
Ultimately fatal McCabe	100 (37.9)	58 (49.2)	1.43 (0.99–2.05)	0.005	1.29 (0.89–1.89)	0.178
Origin of bacteremia						
Catheter-related bloodstream infection	34 (12.9)	5 (4.2)	0.34 (0.14–0.83)	0.017		
Urinary tract infection	96 (36.4)	20 (16.9)	0.41 (0.25–0.66)	<0.001		
Respiratory infection	49 (18.6)	46 (39)	2.25 (1.55–3.26)	<0.001		
Skin and soft tissue infection	12 (4.5)	7 (5.9)	1.26 (0.58–2.69)	0.558		
Intraabdominal infection	29 (11)	16 (13.6)	1.18 (0.69–1.99)	0.537		
Primary or Unknown	39 (14.8)	23 (19.5)	1.38 (0.87–2.17)	0.165		
Other	5 (1.9)	1 (0.8)	0.51 (0.07–3.63)	0.500		
High-risk source	134 (50.8)	93 (78.8)	3.03 (1.96–4.76)	<0.001	2.49 (1.56–3.99)	<0.001
Low-risk source	130 (49.2)	25 (21.2)	0.33 (0.21–0.51)	<0.001		
Baseline illness severity						
Pitt score, m (IQR)	1 (0–2)	4 (1–4)	1.38 (1.28–1.49)	<0.001	1.25 (1.13–1.37)	<0.001
Pitt score ≥ 2	120 (45.5)	86 (72.9)	2.73 (1.82–4.1)	<0.001		
Septic shock	51 (19.3)	52 (44.1)	2.63 (1.83–3.79)	<0.001	1.77 (1.18–2.65)	0.006
ICU admission	44 (16.7)	52 (44.1)	2.99 (2.08–4.3)	<0.001		
Antibiotic management						
Appropriate empirical treatment	114 (43.2)	50 (42.4)	0.98 (0.68–1.41)	0.899		
Appropriate definitive treatment	240 (90.9)	92 (78)	0.42 (0.27–0.65)	<0.001	0.42 (0.27–0.66)	<0.001
Combined antimicrobial therapy	96 (36.4)	54 (45.8)	1.29 (0.9–1.87)	0.157		
XDR PA BSI	81 (30.7)	41 (34.7)	1.13 (0.77–1.65)	0.535	1.14 (0.77–1.69)	0.504

## Supplementary Materials

The following are available online at https://www.mdpi.com/2077-0383/9/2/514/s1, Table S1: Baseline characteristics of patients with high-risk sources of infection, Table S2: Univariate and multivariate Cox model of 14-day all-cause mortality in patients with high-risk sources of infection, Table S3: Univariate and multivariate Cox model of 30-day all-cause mortality in patients with high-risk sources of infection.
