# Supplementary material for: Risk Factors for Mortality among Patients with Pseudomonas aeruginosa Bloodstream Infections: What Is the Influence of XDR Phenotype on Outcomes?
Source: J Clin Med. 2020 Feb 14;9(2):514. doi: 10.3390/jcm9020514 (PMC7074151; doi:10.3390/jcm9020514)
Supplement: Supplementary file 1 [file jcm-09-00514-s001.zip › jcm-705482-supplementary.docx]

**Supplementary material**

**Table S1.** Baseline characteristics of patients with high-risk sources of infection.

**Table S2.** Univariate and multivariate Cox model of 14-day all-cause mortality in patients with high-risk sources of infection.

**Table S3.** Univariate and multivariate Cox model of 30-day all-cause mortality in patients with high-risk sources of infection.

**Table S1. Baseline characteristics of patients with high-risk sources of infection.** Data are presented as *n* (%), unless otherwise specified. Abbreviations m (median), IQR (interquartile range), XDR-PA (extensively drug-resistant *P. aeruginosa*).

| **Variables** | **All Episodes**  **(*n* = 227)** | **Non-XDR PA**  **(*n* = 167)** | **XDR-PA**  **(*n* = 60)** | ***p*-Value** |
| --- | --- | --- | --- | --- |
| Demographic information |  |  |  |  |
| Age in years, m (IQR) | 69 (58–77) | 72 (60–78) | 63 (56–75.75) | **0.043** |
| Male sex | 156 (68.7) | 115 (68.9) | 41 (68.3) | 0.940 |
| Nosocomial acquisition | 145 (63.9) | 93 (55.7) | 52 (86.7) | **<0.001** |
| Underlying condition |  |  |  |  |
| Diabetes Mellitus | 48 (21.1) | 36 (21.6) | 12 (20) | 0.800 |
| Chronic obstructive pulmonary disease | 56 (24.8) | 37 (22.3) | 19 (31.7) | 0.149 |
| Cirrhosis | 15 (6.6) | 12 (7.2) | 3 (5) | 0.764 |
| Hemodialysis | 10 (4.4) | 9 (5.4) | 1 (1.7) | 0.298 |
| Hematology malignancy | 39 (17.2) | 20 (12) | 19 (31.7) | **0.001** |
| Solid tumor malignancy | 77 (33.9) | 63 (37.7) | 14 (23.3) | **0.043** |
| Neutropenia | 55 (24.2) | 39 (23.4) | 16 (26.7) | 0.607 |
| Charlson comorbidity index, m (IQR) | 4 (2–6) | 4 (2–6) | 3 (2–6) | 0.548 |
| McCabe Score |  |  |  |  |
| Non-fatal McCabe | 63 (27.8) | 46 (27.5) | 17 (28.3) | 0.907 |
| Rapidly fatal McCabe | 69 (30.4) | 51 (30.5) | 18 (30) | 0.938 |
| Ultimately fatal McCabe | 95 (41.9) | 70 (41.9) | 25 (41.7) | 0.973 |
| Source of infection |  |  |  |  |
| Respiratory infection | 95 (41.9) | 67 (40.1) | 28 (46.7) | 0.378 |
| Soft skin tissue infection | 19 (8.4) | 15 (9) | 4 (6.7) | 0.579 |
| Intraabdominal infection | 45 (19.8) | 36 (21.6) | 9 (15) | 0.275 |
| Primary or Unknown | 62 (27.3) | 45 (26.9) | 17 (28.3) | 0.836 |
| Other | 6 (2.6) | 4 (2.4) | 2 (3.3) | 0.656 |
| Baseline illness severity |  |  |  |  |
| Pitt score, m (IQR) | 2 (1–4) | 2 (1–4) | 3 (1–4) | **0.01** |
| Pitt score ≥ 2 | 133 (58.6) | 92 (55.1) | 41 (68.3) | 0.074 |
| Septic shock | 72 (31.7) | 48 (28.7) | 24 (40) | 0.108 |
| Antibiotic management |  |  |  |  |
| Appropriate empirical treatment | 105 (46.3) | 98 (58.7) | 7 (11.7) | **<0.001** |
| Appropriate definitive treatment | 192 (84.6) | 142 (85) | 50 (83.3) | 0.755 |
| Combined antimicrobial therapy | 106 (46.7) | 70 (41.9) | 36 (60) | **0.016** |
| All-cause mortality |  |  |  |  |
| Day 14 | 74 (32.6) | 51 (30.5) | 23 (38.3) | 0.269 |
| Day 30 | 93 (41) | 63 (37.7) | 30 (50) | 0.097 |

**Table S2. Univariate and multivariate Cox analyses of 14-day all-cause mortality in patients with high-risk sources of infection.** Data are presented as *n* (%), unless otherwise specified. Abbreviation: HR (Hazard ratio), CI (confidence interval), m (median), IRQ (interquartile range), XDR-PA (extensively drug-resistant *P. aeruginosa*), BSI (Bloodstream Infection).

| **Variables** | **Alive**  **(*n* = 155)** | **Death**  **(*n* = 74)** | **Crude HR**  **(95% CI)** | ***p*-Value** | **HR Multivariate**  **(95% CI)** | ***p*-Value** |
| --- | --- | --- | --- | --- | --- | --- |
| Demographic information |  |  |  |  |  |  |
| Age in years, m (IQR) | 69 (57–77.5) | 69 (60–75) | 1.01 (0.99–1.02) | 0.385 | 1.01 (0.99–1.03) | 0.211 |
| Male sex | 101 (66) | 55 (74.3) | 1.36 (0.81–2.29) | 0.247 | 1.25 (0.73–2.13) | 0.412 |
| Nosocomial acquisition | 97 (63.4) | 48 (64.9) | 1.05 (0.65–1.69) | 0.839 |  |  |
| Underlying condition |  |  |  |  |  |  |
| Diabetes Mellitus | 34 (22.2) | 14 (18.9) | 0.86 (0.48–1.54) | 0.619 |  |  |
| Chronic obstructive pulmonary disease | 37 (24.3) | 19 (25.7) | 1.06 (0.63–1.79) | 0.827 |  |  |
| Cirrhosis | 8 (5.2) | 7 (9.5) | 1.63 (0.75–3.55) | 0.219 |  |  |
| Hemodialysis | 8 (5.2) | 2 (2.7) | 0.55 (0.13–2.23) | 0.398 |  |  |
| Hematology malignancy | 24 (15.7) | 15 (20.3) | 1.21 (0.68–2.13) | 0.515 |  |  |
| Solid tumor malignancy | 51 (33.3) | 26 (35.1) | 1.05 (0.65–1.7) | 0.834 |  |  |
| Neutropenia | 38 (24.8) | 17 (23) | 0.89 (0.52–1.54) | 0.692 |  |  |
| Charlson comorbidity index, m (IQR) | 4 (2–6) | 4 (2–6) | 1.02 (0.94–1.1) | 0.613 |  |  |
| McCabe Score |  |  | 1.3 (0.97–1.74) | 0.077 |  |  |
| Non-fatal McCabe | 47 (30.7) | 16 (21.6) | 0.72 (0.41–1.24) | 0.236 |  |  |
| Rapidly fatal McCabe | 49 (32) | 20 (27) | 0.81 (0.48–1.35) | 0.408 |  |  |
| Ultimately fatal McCabe | 57 (37.3) | 38 (51.4) | 1.54 (0.98–2.43) | 0.063 | 1.44 (0.91–2.31) | 0.123 |
| Origin of bacteremia |  |  |  |  |  |  |
| Respiratory infection | 60 (39.2) | 35 (47.3) | 1.31 (0.83–2.06) | 0.252 |  |  |
| Soft skin tissue infection | 13 (8.5) | 6 (8.1) | 0.94 (0.41–2.17) | 0.885 |  |  |
| Intraabdominal infection | 35 (22.9) | 10 (13.5) | 0.59 (0.3–1.14) | 0.116 |  |  |
| Primary or Unknown | 40 (26.1) | 22 (29.7) | 1.16 (0.7–1.91) | 0.566 |  |  |
| Other | 5 (3.3) | 1 (1.4) | 0.46 (0.06–3.35) | 0.448 |  |  |
| Baseline illness severity |  |  |  |  |  |  |
| Pitt score, m (IQR) | 2 (0–3.5) | 4 (1–4) | 1.26 (1.14–1.39) | **<0.001** | 1.21 (1.08–1.36) | **0.001** |
| Pitt score ≥ 2 | 78 (51) | 55 (74.3) | 2.48 (1.47–4.18) | **0.001** |  |  |
| Septic shock | 36 (23.5) | 36 (48.6) | 2.57 (1.63–4.07) | **<0.001** | 2.16 (1.31–3.56) | **0.003** |
| Antibiotic management |  |  |  |  |  |  |
| Appropriate empirical treatment | 75 (49) | 30 (40.5) | 0.76 (0.48–1.21) | 0.246 |  |  |
| Appropriate definitive treatment | 139 (90.8) | 53 (71.6) | 0.34 (0.2–0.57) | **<0.001** | 0.42 (0.24–0.71) | **0.001** |
| Combined antimicrobial therapy | 77 (50.3) | 29 (39.2) | 0.67 (0.42–1.07) | 0.092 | 0.56 (0.33–0.93) | **0.027** |
| XDR-PA BSI | 37 (24.2) | 23 (31.1) | 1.28 (0.78–2.08) | 0.338 | 1.12 (0.67–1.86) | 0.667 |

**Table S3. Univariate and multivariate Cox analyses of 30-day all-cause mortality in patients with high-risk sources of infection.** Data are presented as *n* (%), unless otherwise specified. Abbreviation: HR (Hazard ratio), CI (confidence interval), m (median), IQR (interquartile range), XDR-PA (extensively drug-resistant *P. aeruginosa*), BSI (Bloodstream Infection).

| **Variables** | **Alive**  **(*n* = 134)** | **Death**  **(*n* = 93)** | **Crude HR**  **(95% CI)** | ***p*-Value** | **HR Multivariate**  **(95% CI)** | ***p*-Value** |
| --- | --- | --- | --- | --- | --- | --- |
| Demographic information |  |  |  |  |  |  |
| Age in years, m (IQR) | 68 (57–77) | 70 (62–77) | 1.01 (0.99–1.02) | 0.385 | 1.01 (0.99–1.03) | 0.102 |
| Male sex | 86 (64.2) | 70 (75.3) | 1.37 (0.81–2.29) | 0.247 | 1.25 (0.78–2.01) | 0.361 |
| Nosocomial acquisition | 80 (59.7) | 65 (69.9) | 1.05 (0.65–1.69) | 0.839 |  |  |
| Underlying condition |  |  |  |  |  |  |
| Diabetes Mellitus | 29 (21.6) | 19 (20.4) | 0.86 (0.42–1.54) | 0.619 |  |  |
| Chronic obstructive pulmonary disease | 31 (23.1) | 25 (27.2) | 1.06 (0.63–1.79) | 0.827 |  |  |
| Cirrhosis | 8 (6) | 7 (7.5) | 1.63 (0.75–3.55) | 0.219 |  |  |
| Hemodialysis | 8 (6) | 2 (2.2) | 0.55 (0.14–2.22) | 0.398 |  |  |
| Hematology malignancy | 21 (15.7) | 18 (19.4) | 1.21 (0.69–2.13) | 0.515 |  |  |
| Solid tumor malignancy | 46 (34.3) | 31 (33.3) | 1.05 (0.65–1.69) | 0.834 |  |  |
| Neutropenia | 37 (27.6) | 18 (19.4) | 0.89 (0.52–1.54) | 0.692 |  |  |
| Charlson comorbidity index, m (IQR) | 4 (2–6) | 4 (2–6) | 1.02 (0.94–1.1) | 0.613 |  |  |
| McCabe |  |  | 1.29 (0.97–1.74) | 0.077 |  |  |
| Non-fatal McCabe | 42 (31.3) | 21 (22.6) | 0.72 (0.41–1.25) | 0.236 |  |  |
| Rapidly fatal McCabe | 42 (31.3) | 27 (29) | 0.81 (0.49–1.34) | 0.408 |  |  |
| Ultimately fatal McCabe | 50 (37.3) | 45 (48.4) | 1.54 (0.98–2.43) | 0.063 | 1.28 (0.84–1.95) | 0.245 |
| Source of infection |  |  |  |  |  |  |
| Respiratory infection | 49 (36.6) | 46 (49.5) | 1.31 (0.83–2.06) | 0.252 |  |  |
| Soft skin tissue infection | 12 (9) | 7 (7.5) | 0.94 (0.41–2.17) | 0.885 |  |  |
| Intraabdominal infection | 29 (21.6) | 16 (17.2) | 0.59 (0.3–1.14) | 0.116 |  |  |
| Primary or Unknown | 39 (29.1) | 23 (24.7) | 1.16 (0.7–1.91) | 0.566 |  |  |
| Other | 5 (3.7) | 1 (1.1) | 0.47 (0.06–3.35) | 0.448 |  |  |
| Baseline illness severity |  |  |  |  |  |  |
| Pitt score, m (IQR) | 1 (0–3) | 4 (2–4) | 1.26 (1.14–1.39) | **<0.001** | 1.23 (1.11–1.36) | **<0.001** |
| Pitt score ≥ 2 | 63 (47) | 70 (75.3) | 2.48 (1.47–4.18) | **0.001** |  |  |
| Septic shock | 27 (20.1) | 45 (48.4) | 2.58 (1.63–4.07) | **<0.001** | 1.97 (1.27–3.06) | **0.003** |
| Antibiotic management |  |  |  |  |  |  |
| Appropriate empirical treatment | 69 (51.5) | 36 (38.7) | 0.76 (0.48–1.21) | 0.246 |  |  |
| Appropriate definitive treatment | 121 (90.3) | 71 (76.3) | 0.34 (0.21–0.57) | **<0.001** | 0.41 (0.25–0.67) | **<0.001** |
| Combined antimicrobial therapy | 64 (47.8) | 42 (45.2) | 0.85 (0.57–1.29) | 0.451 |  |  |
| XDR-PA BSI | 30 (22.4) | 30 (32.3) | 1.39 (0.89–2.14) | 0.140 | 1.18 (0.76–1.85) | 0.457 |
